# Privacy-Preserved Fall Detection Method with Three-Dimensional Convolutional Neural Network Using Low-Resolution Infrared Array Sensor

**DOI:** 10.3390/s20205957

**Published:** 2020-10-21

**Authors:** Shigeyuki Tateno, Fanxing Meng, Renzhong Qian, Yuriko Hachiya

**Affiliations:** 1 Graduate School of Information Production and Systems, Waseda University, Kitakyushu 808-0135, Japan; mengfanxing@akane.waseda.jp (F.M.); qian_renzhong@suou.waseda.jp (R.Q.); 2 School of Health Sciences, University of Occupational and Environmental Health, Kitakyushu 807-8555, Japan; hachiya@med.uoeh-u.ac.jp

**Keywords:** human motion detection, falling, infrared array sensor, privacy protection, three-dimensional convolutional neural network

## Abstract

Due to the rapid aging of the population in recent years, the number of elderly people in hospitals and nursing homes is increasing, which results in a shortage of staff. Therefore, the situation of elderly citizens requires real-time attention, especially when dangerous situations such as falls occur. If staff cannot find and deal with them promptly, it might become a serious problem. For such a situation, many kinds of human motion detection systems have been in development, many of which are based on portable devices attached to a user’s body or external sensing devices such as cameras. However, portable devices can be inconvenient for users, while optical cameras are affected by lighting conditions and face privacy issues. In this study, a human motion detection system using a low-resolution infrared array sensor was developed to protect the safety and privacy of people who need to be cared for in hospitals and nursing homes. The proposed system can overcome the above limitations and have a wide range of application. The system can detect eight kinds of motions, of which falling is the most dangerous, by using a three-dimensional convolutional neural network. As a result of experiments of 16 participants and cross-validations of fall detection, the proposed method could achieve 98.8% and 94.9% of accuracy and F1-measure, respectively. They were 1% and 3.6% higher than those of a long short-term memory network, and show feasibility of real-time practical application.

## 1. Introduction

With the trend of global aging, various social problems have emerged one after another such as economic, political, and social development issues; population projections; and so on [1]. A large number of countries are stepping up research on population aging [2]. As the number of elderly people in hospitals and nursing homes surges, expectations for advanced systems that accurately alert them to dangerous motions and emergencies in real-time are increasing [3], and the combination of security care and intelligent management can solve this problem. By developing artificial intelligence and leveraging big data, many everyday applications can be automated. [4]. Although some nursing systems that focus on monitoring and physical condition measurement have been developed, there are still some requirements that have not been significantly satisfied, which shows that this research field has broad application prospects.

There are various forms of motion that need to be detected. Of these, falling is one of the most dangerous ones and should be paid attention to [5]. It might cause serious consequences or severe sequelae such as fracture, soft tissue injury, and psychological trauma, making an impact on the mental and physical health of elderly. According to the World Health Organization (WHO), falls are the second leading cause of accidental deaths, with approximately 646,000 deaths each year, especially those over the age of 65, and 37.3 million falls occur each year and require medical attention [6].

From the view of healthcare, shortage of medical personnel is another problem. The WHO reported that the global health workforce was 43.5 million in 2013 and it would be 67.3 million in 2030, however a shortage of the global health workforce was 17.4 million in 2013, and it would be 14.5 million in 2030 [7,8]. Moreover, the WHO stated that the population of over 60 would be more than 22% of the total of world population [9]. These facts showed that the shortage of medical staff has been unsolved.

The increase in falls with aging is inevitable, and proper health care is important as well as prevention. It has a fatal adverse effect on the physical and mental health of the elderly and also increases the burden on families and society; therefore, it has become a central topic in geriatrics [10,11,12]. To protect the physical and mental safety of the elderly and improve their living standards, it is necessary to have a system in place to take certain measures to track and analyze their daily motions. In the event of a fall, the emergency information should be sent to community caregivers or their family members as quickly as possible in order to ensure their safety, shorten the rescue time, and give them proper treatment [13].

In this research, from the perspective of versatility and privacy, a fall detection method based on one low-resolution infrared array sensor is proposed. This sensor is operational in the dark, and the low resolution allows privacy to be preserved. The target of this research was to achieve the aims of human motion detection and send an alarm message when a dangerous situation occurred in a hospital or a nursing room since these are the places where the elderly particularly need to be cared for.

The main contributions of this study are as follows:

1. We proposed a motion detect method which can mainly detect falling in consideration of privacy preservation for elderly people using low resolution infrared array sensor. In our proposed system, one sensor can cover a 4 × 5 m area for detection, and the area can be expanded by using more than one independent sensor. It means that our system has high flexibility and scalability.
2. We proposed to adapt a three-dimensional convolutional neural network as a suitable classifier for the proposed system. It could detect an occurrence of falling with very high accuracy of 98.8% and F1-measure of 94.9% in real-time, which were 1% and 3.6% higher than those of a long short-term memory network, respectively. It means that the proposed system could achieve construction of an alert system for the health care and support caretakers.

This paper is organized as follows: Section 2 provides an overview of relate work about fall detection. Section 3 shows materials and methods of proposed system. Section 4 explains detail of experiments, and Section 5 shows the result of the experiments. After that, Section 6 discusses the results. Finally, Section 7 concludes this study.

## 2. Related Work

There mainly exist two kinds of fall detection systems: systems based on portable devices and systems based on external sensing devices. First, portable or wearable device-based methods require sensors such as accelerometers and gyroscopes to be attached to parts of the human body, and these sensors are used to detect the acceleration and angular velocity of specific parts of the body [14]. Mathie et al. used an accelerometer attached to the waist to detect falls [15]. The movement of the human body from standing to falling causes acceleration to increase suddenly, so the fall can be judged accordingly. Bianchi et al. introduced a barometric pressure sensor as a tool for height measurement to improve the existing human fall detection technology based on accelerometers. The device is worn on the waist, records the acceleration and barometric pressure data, then processes the data, and finally uses the trained hierarchical decision tree model to perform human fall detection [16]. Haoyu et al. considered the risk level of a fall including the direction of the fall and whether it was supported by a hand by constructing a three-layer structure with machine learning algorithms based on wearable sensors [17].

Of the methods based on external sensing devices, camera-based methods take advantage of the rapid development of image processing technology. Computer vision-based motion detection takes a video of a person’s motion with an optical camera and uses advanced image processing algorithms to determine whether there is a frame with a motion feature to detect [18]. De Miguel et al. developed a fall detection system based on a camera for the elderly that applies various algorithms to extract better features and used a K-nearest neighbors algorithm to achieve recognition [19]. Yu et al. used an enhanced one-class support vector machine as a recognition algorithm and obtained features including the differences of barycenter position and orientation of a person over a time period as input [20]. Merrouche et al. used a depth camera to detect a fall by combining human shape and movement. They analyzed shape features and movement of the human and tracked the human head through particle filter while expressing movement by the covariance of the center of mass distance over time [21]. There are other types of external sensing devices. Chelli et al. and Wang et al. used Wi-Fi to detect human motions with a support vector machine (SVM) [22,23]. Mokhtari et al. developed a fall detection system based on an ultra-wide band (UWB) radar with an SVM [24]. Sadreazami et al. and Liang Ma et al. also used a UWB radar for fall detection with a convolutional neural network (CNN) [25,26].

During actual application, different living environments and their limitations should be considered to ensure the convenience, reliability, and practicability of the system. For instance, wearable devices inevitably lead to decreased comfort. Moreover, in private areas such as restrooms and bathrooms, privacy should be protected, which means that the application of an optical camera is restricted in such situations. When designing the system, these problems should be considered carefully [27].

According to a survey, infrared sensors have been used for human detection [28], target tracking [29], and motion recognition [30]. High-resolution sensors and infrared sensor arrays are commonly used in motion detection but are insufficient for privacy protection and have a high price. Some studies which focused on fall detection using low resolution infrared sensor are summarized in Table 1. The accuracy of each study is discussed in Section 7. Among them, Tao et al. proposed a privacy-preserved fall detection system using an infrared ceiling sensor network [31], which was similar to our proposed system. The resolution of the sensors used is quite low, just 4 × 5 pixels, so they adopted a sensor network, which made the system complex.

## 3. Materials and Methods

This section explains the sensor used in this study, system construction, system flow chart of proposed methods, data processing of thermo images, the method of target existence detection and positioning, and two kinds of classifiers selected in this study.

### 3.1. Infrared Array Sensor

An infrared sensor is a kind of sensor that uses the physical properties of infrared light to make measurements. Infrared sensors are usually used for non-contact temperature measurement, gas composition analysis, and non-destructive testing, and are widely used in the fields of medicine, military, space technology, and environmental engineering [42].

Commonly, infrared sensors are divided into two types: near-infrared (NIR) and far-infrared (FIR). Compared with the NIR sensor, the FIR sensor has a stronger anti-interference ability. Compared with other infrared sensors such as passive infrared detectors and single-point infrared sensors, infrared array sensors can provide more comprehensive information including position information, movement states, surface temperature, and others [43].

In this research, from the perspective of versatility and privacy, one detection method based on a low-resolution infrared array sensor is proposed. In the proposed motion detection system, the low-resolution infrared array sensor can protect the privacy of the elderly, which means, it can be used in some private places such as locker rooms and restrooms. This method can effectively recognize elderly people’s fundamental motions and detect their emergencies.

**Figure 1:**
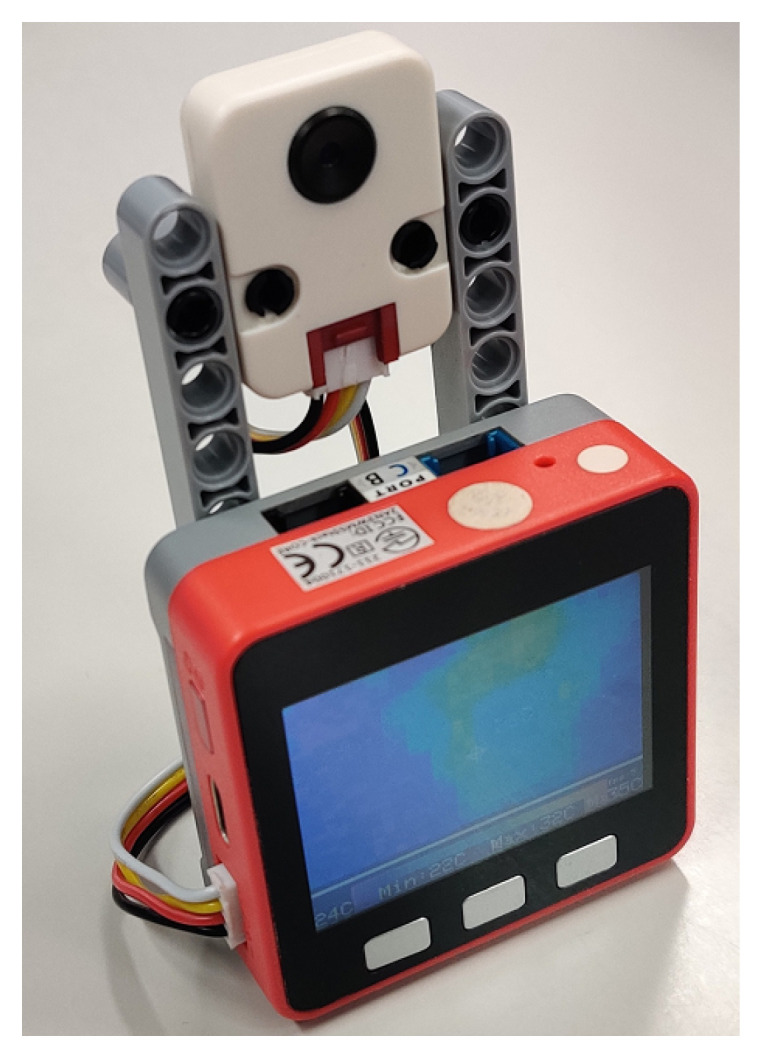
The M5Stack and the infrared array sensor.

A far infrared thermal sensor named MLX90640, manufactured by Melexis, was applied in this research [44]. The 32 × 24 thermopile elements in the sensor can detect infrared radiation of objects in the detection area and return a low-resolution thermal image that reflects the temperatures of the objects [32]. The normal working temperature of the sensor varies from −40 to 85 °C, which means that this sensor may perform well in all common conditions. The sampling rate of the sensor is programmable up to 64 Hz, which means that this sensor can be used for real-time detection [45,46]. An I2C compatible digital interface is provided for data transmission with a transmission frequency of up to 1 MHz. In this research, the sensor was connected to a micro-controller unit, M5Stack including a Wi-Fi module, through a GROVE I2C port as shown in Figure 1. The data collected by the M5Stack can be sent to a PC with Wi-Fi via routers in a separate location such as a nurse station or a staff operation room in a remote monitoring system. The data gathered in the PC was continuously processed to detection a fall, and when detected, the system announce an alert to staff.

In this system, the acquisition frequency of the sensor was set to 15 Hz considering the data size to be processed because the data processing time can be relatively short.

**Figure 2:**
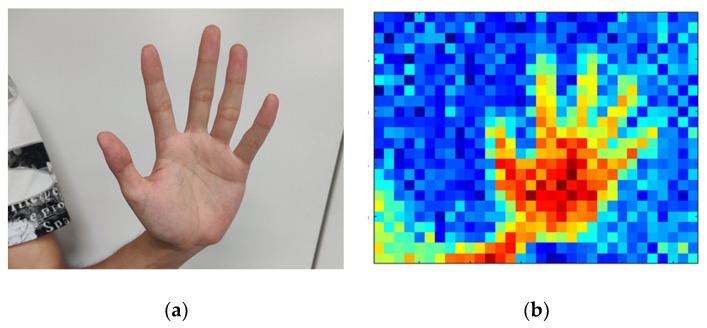
Example of far-infrared imaging: (**a**) original image; (**b**) far-infrared imaging.

An example of an infrared array image is shown in Figure 2. Here, Figure 2a shows the real hand image and Figure 2b shows the RGB colored infrared array image. Originally, the infrared image is grayscale (8 bits); however, here it is shown in color for clear and easy viewing.

### 3.2. System Flow Chart of the Propsed Method

**Figure 3:**
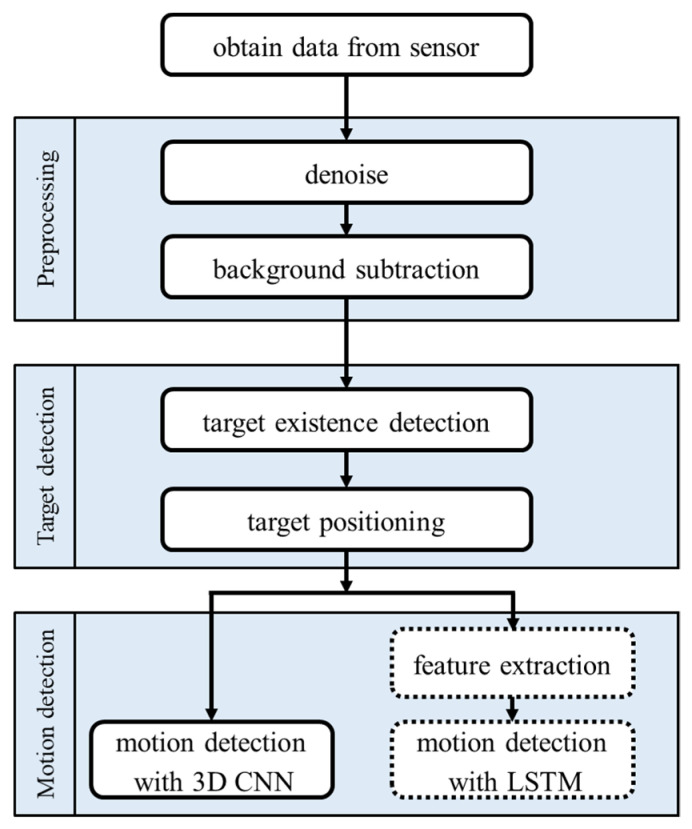
Flow chart of the proposed system.

The flow chart of the proposed motion detection system is shown in Figure 3. The processing methods are mainly divided into three parts: preprocessing, target detection, and motion detection. In the first part, the raw data frames obtained from the sensor are denoised, and then separated into two parts, background and objects. The background can be removed to extract an area of the objects. In the next part, methods of target presence detection and target location are used for target detection. Finally, in the last part, a three-dimensional convolutional neural network (3D CNN) [47] is applied to motion detection to deal with the time series image data. A long short-term memory (LSTM) network with feature extraction is also applied for comparison.

### 3.3. Data Preprocessing

After the data frames are collected from the sensor, preprocessing including denoising and background subtraction is executed. In consideration of the noise distribution. Gaussian filtering, which is a linear smoothing filter, has been commonly used to reduce noise in image processing [48], was chosen. In this research, the data size and noise intensity were considered when determining the filter; therefore, the smallest size filter shown in Equation (1) is sufficient.

(1) $$Gaussian\;filter=\;\left[\begin{array}{ccc} 1/16 & 1/8 & 1/16\\ 1/8 & 1/4 & 1/8\\ 1/16 & 1/8 & 1/16 \end{array}\right].\;$$

**Figure 4:**
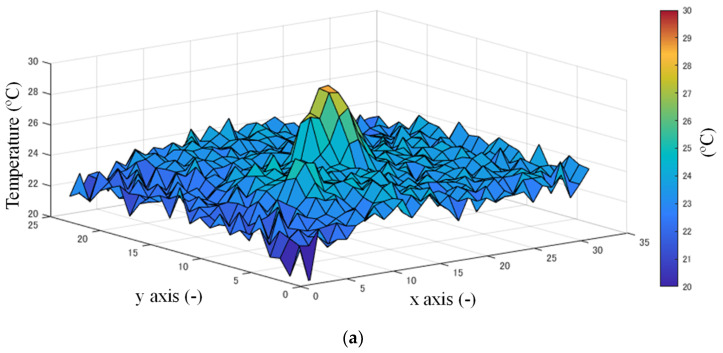

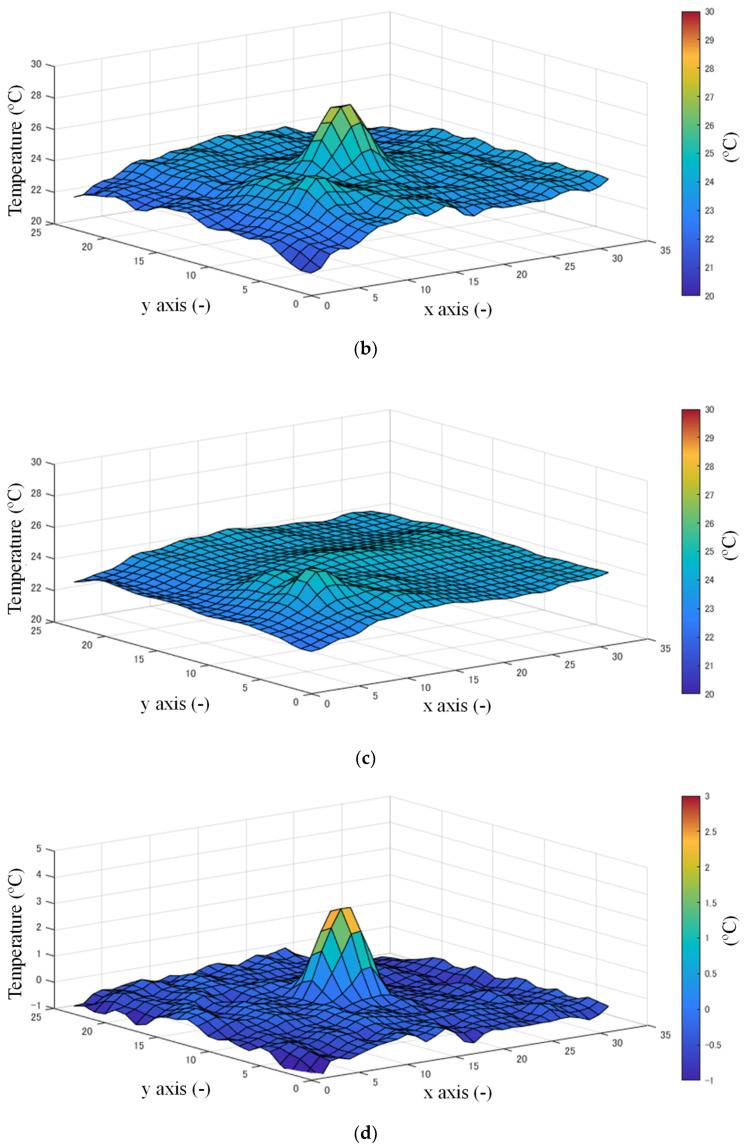
Example of temperature distribution: (**a**) data before Gaussian filtering; (**b**) data after Gaussian filtering; (**c**) data of background; (**d**) data after background subtraction.

Figure 4a shows a typical frame of raw data where the target exists. Data after the Gaussian filtering is shown in Figure 4b. After denoising, background subtraction is executed to extract the target. It is a common method used to record some frames when there is no target in the area, and these data are regarded as the stable background.

In real applications such as wards of a hospital, there is usually some electronic equipment that heats when operational, such as heart rate monitor devices, which may be detected and judged as a human and cause false detection. To deal with this problem, background subtraction was applied. The distribution of the data of the background is shown in Figure 4c.

**Figure 5:**
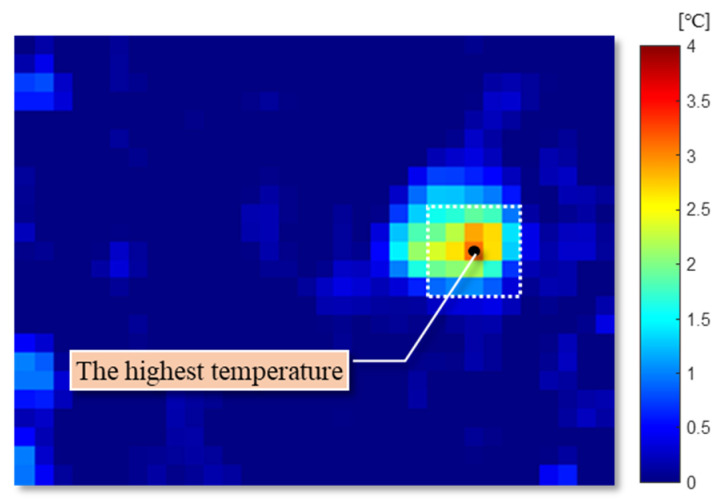
The human detection in the infrared data.

In general, non-human items do not move frequently, so we collected a relatively long series of data and used the average as the initial background, renewing by

(2) $$BG_{i}=\alpha \cdot BG_{i-1}\;+\;\left(1-\alpha \right)\cdot FG_{i},\;$$

where $BG_{i}$ means the background in the *i*-th frame and $FG_{i}$ means the foreground data of the *i*-th frame. The weight α is a constant decided by the application environment. This background subtraction and renewing method shows suitable performance without much calculation cost. Figure 4d shows the data of Figure 5 after background subtraction.

### 3.4. Target Existence Detection and Positioning

After the background subtraction, firstly, the target detection is performed for each frame. It can be assumed that temperatures of a human are higher than those of the environment and emit more heat radiation, so the human image consists of spots with higher temperatures in the thermal image. In the application environments of hospitals and nursing rooms, a few moving non-human items can emit a large amount of heat radiation, so the aim of human detection can be simplified as the detection of larger heating spots. First, the local highlighted temperature pixel and the 5 × 5 neighborhood pixels are selected. Then, the selected pixels with temperatures at least 1 °C higher than the background are marked. When the size of a marked area is more than or equal to $N_{H}$ pixels, it indicates that this area can be detected as a human [44], as follows:

(3) $$human\;detecton=\;\{\begin{array}{c} \;1\;\left(yes\right)\;if\;maked\;pixcel\;\geq N_{H},\\ \;0\;\left(no\right)\;if\;maked\;pixcel<\;N_{H}, \end{array}\;$$

where, $N_{H}$ is a threshold for human detection. In Figure 5, the highest temperature pixel of the detected target is shown as a black point. Here, $N_{H}$ is also decided based on the target size in the system environment.

**Figure 6:**
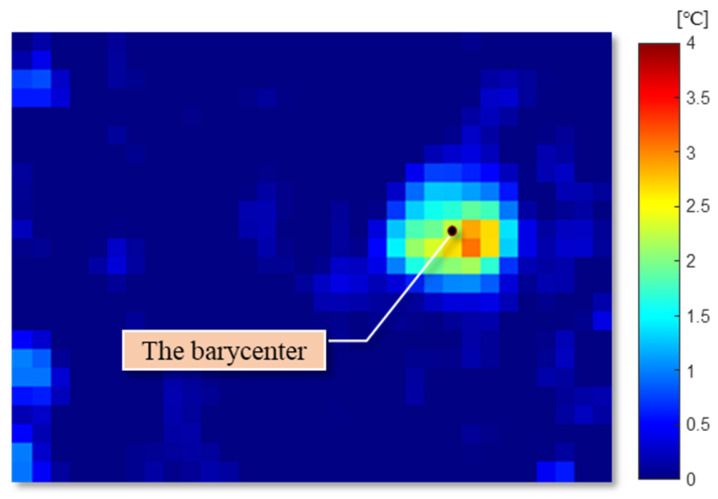
The adjusted center point of the target in the infrared data.

The representative pixel with the highest value is not always the middle point of a human, because it is influenced by clothes, gestures, and noise. To correctly express the position of the target and obtain better performance, the target position should be corrected. The barycenter of the neighborhood in the local highlighted pixels is calculated by Equation (4).

(4) $$\left(x_{c\;},\;y_{c}\right)\;=\;\left(\frac{\sum_{x,y}a_{x,y}x}{\sum_{x,y}a_{x,y}}\;,\;\frac{\sum_{x,y}a_{x,y}y}{\sum_{x,y}a_{x,y}}\;\right),$$

where $\left(x_{c},\;y_{c}\right)$ is the coordinate of the barycenter in this frame of the image, $a_{x,y}$ is the value on point (x,y). This process is repeated with the renewed middle point and the same neighborhood size until the barycenter stop moving. Figure 6 shows the adjusted center point of the target with a black point [45]. The barycenters obtained from sequential frames can be used to detect a moving direction, a moving distance, and a speed of the target in this system.

The motion detection is based on the result of the target tracking. When there are multiple targets in the frame, motion detection can be performed accordingly, and when one target is judged to disappear from the area, the motion detection for the target is stopped. 

### 3.5. Classifiers Used in the System

#### 3.5.1. Three-Dimensional Convolutional Neural Network

A traditional two-dimensional convolutional neural network (2D CNN) for video images with continuous frames can easily lose the information on the target time axis, resulting in low recognition accuracy. To address this problem, a method based on a 3D CNN is proposed [49]. Three-dimensional CNN has received considerable attention over the years for use in consecutive frames understanding. The main reason for its success is the effective extraction of spatio-temporal features from consecutive frames. The 3D convolution kernel is used to extract temporal and spatial features to capture the motion information of the object [50,51].

**Figure 7:**
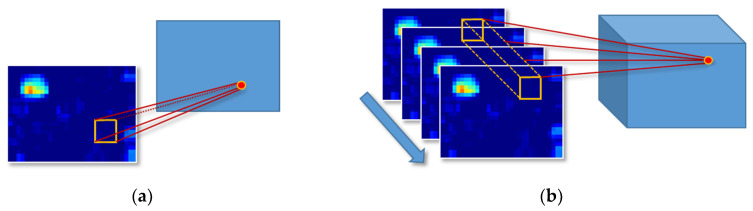
A 2D convolution and a 3D convolution: (**a**) description of the 2D convolutional neural network (CNN); (**b**) description of the 3D CNN.

In Figure 7a, the traditional 2D CNN uses a 2D convolution kernel to convolve the image. The time and space feature information in the continuous figures can be better dealt with by using a 3D CNN. The 3D convolutional neural network includes a convolutional layer, a pooling layer, a fully connected layer, and a softmax layer. The 3D convolutional layer expands the dimensions of the convolutional neural network based on the basis of the 2D convolutional neural network. The size of the convolution kernel and the filter size of the pooling layer in each layer of the network structure are all upgraded to three dimensions. The output of the convolutional layer is cube data. In the fully connected layer, neurons are connected to all neurons in the adjacent layer. The input of the fully connected layer converts the feature space to a neuron vector and then uses matrix multiplication to flatten the input feature vector. The input of the previous layer flattens the convolved 3D feature vector to a neuron vector. The final output layer is the softmax layer, and the last neural vector will calculate the probability of each category. In Figure 7b, the 3D CNN uses a 3D convolution kernel to perform convolution operations on the image sequence (video) [52,53]. The time dimension of the convolution operation in the figure is three, that is, the convolution operation on three consecutive frames of images. The 3D convolution in Figure 7b forms a cube by stacking multiple consecutive frames, and then the convolution operation is performed using the cube [54]. In this structure, each feature map in the convolutional layer is related to multiple adjacent consecutive frames in the previous layer, which is how it can capture motion information. The value of a certain position of a convolution map is obtained by convolving the local receptive field of the same position of several consecutive frames in the previous layer [55].

**Figure 8:**
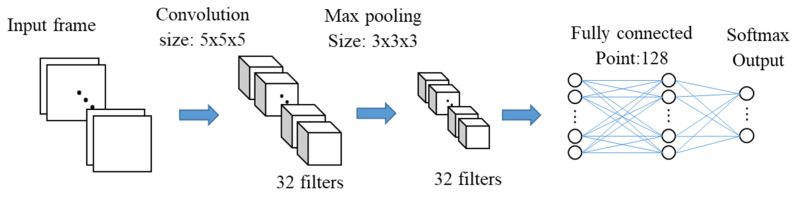
Flow chart of the 3D convolutional neural network (CNN).

The flow chart of the 3D CNN classifiers applied in this system is shown in Figure 8. In this research, the 3D CNN has a structure as follows:

- A convolutional layer with 32 filters and 5 × 5 × 5 cube size for each filter;
- A ReLU layer to complete the activation;
- A max-pooling layer with a cube size of 3 × 3 × 3;
- A fully connected layer with 128 points;
- A ReLU layer with 50% dropout to avoid overfitting;
- A fully connected layer with 8 points;
- A softmax layer and a classification layer.

Each parameter is decided according to the complexity of the target and the number of classes [46]. The effect of convolutional layers is convolving the input and the result is passed to the next layer. The number of free parameters can be manifestly reduced by the convolution operation, allowing it possible that the network to be deeper with fewer parameters. The vanishing gradient and exploding gradient problems that occur during backpropagation in traditional neural networks are prevented by applying regularized weights over fewer parameters. Pooling layers can combine the neuron clusters outputs into a single neuron just at one layer to reduce the dimensions of the data in the next layer, reducing the risk of overfitting. In our research, a pre-test was performed to choose the best size of convolution kernel and pooling window by comparing detection accuracy. In the pre-test, 50 fall data were gathered from each of the 10 participants as a dataset, and 80% of the dataset was used as training, and 20% was used as testing.

#### 3.5.2. A Long Short-Term Memory

An LSTM network has a structure used to process sequence data that changes with time, space, and other factors. The LSTM network can solve the long-term dependence of the data model, that is, the time-based dependence of data. In continuous frame data, the LSTM network predicts the time-series sequence data of the future frame based on the previous sequence data, and expresses the spatio-temporal characteristics in a vector, as input of the cascaded an LSTM unit. The output of the LSTM unit contains the spatio-temporal information. The LSTM network adds a forgetting unit to the structure of a recurrent neural network (RNN) to solve the problem of gradient disappearance and gradient explosion during long sequence training, making it perform better in longer sequences [56]. The LSTM network replaces hidden and output layer nodes with memory cells based on the RNN, which consists of input gates, forget gates, and output gates to control the update of the memory state vectors [57,58]. It can alleviate the problem of gradient loss during recursive neural network training.

**Figure 9:**
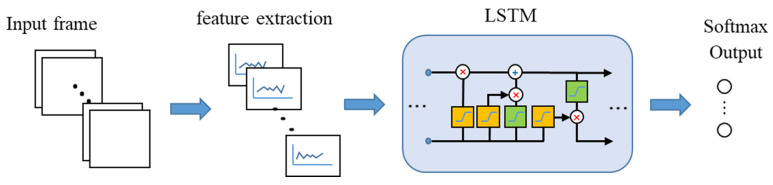
Flow chart of the long short-term memory (LSTM) network.

Although the nature of the LSTM network originally takes advantage in prediction problems, some studies used the LSTM network or hybrid methods with it as a classifier for time series data of human motions [34,59]. In this research, an LSTM network was used with 128 hidden neurons, 6 dimensions at each time step, and a fully connected layer with 8 hidden neurons. Here, the time step size is the number of frames. Considering the characteristics of human motions, six features were extracted and input into the LSTM network. The flow chart of the LSTM network is shown in Figure 9.

Before obtaining features, the Otsu method is applied to separate the target from the complete picture [60]. The Otsu method is an efficient algorithm for binarizing images automatically obtaining a threshold from a bimodal situation, which divides the image into two parts, the background and the target, according to the grayscale characteristics of the image. After that, six features are extracted for detection: a moving distance, an area size, a change rate of area size of the target, the highest temperature, the average temperature, and a directional distribution ratio.

Among them, the change rate of area size is calculated by dividing the area size of the target in the frame by that of the previous frame as follows:

(5) $$change\;rate\;of\;area\;size=\frac{{\mathrm{Area}}_{n}}{{\mathrm{Area}}_{n-1}}$$

**Figure 10:**
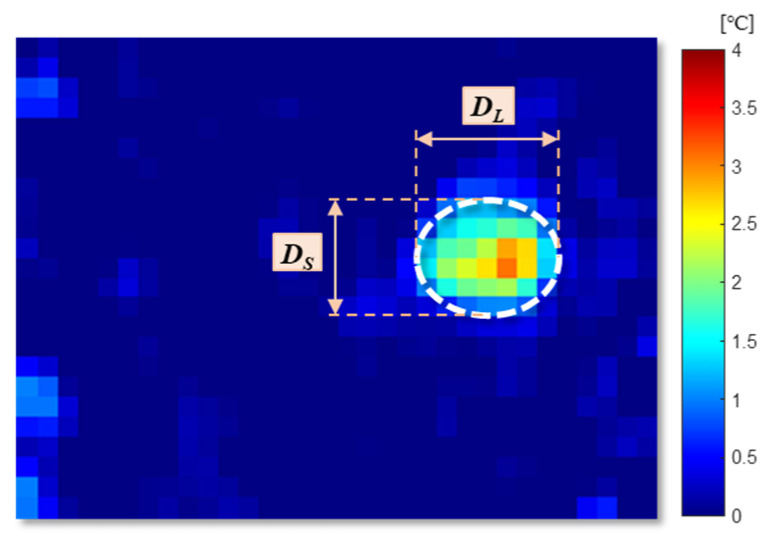
Image description of $D_{L}$ and $\;D_{S}$.

The directional distribution ratio is calculated from directional distribution of the target. This is a spatial statistics algorithm that calculates lengths of the ellipse in the *x* and *y* directions by centering on the average center of the element space distribution, thereby defining the axis of the ellipse containing the element distribution [61]. This feature is defined as the ratio between the longer axis and the short one as shown in Figure 10.

The equation to calculate the directional distribution ratio is as follows:

(6) $$directional\;distribution\;ratio=\;\frac{\;D_{L\;}}{\;D_{S\;}}\;,$$

where $D_{L}$ and $D_{S}$ represent the length of the longer axis and shorter axis of the detected ellipse, respectively.

## 4. Experiment

This section explains the experimental environment and setting, dataset of the target motions, and experimental ethics.

### 4.1. Experiment Environment

**Figure 11:**
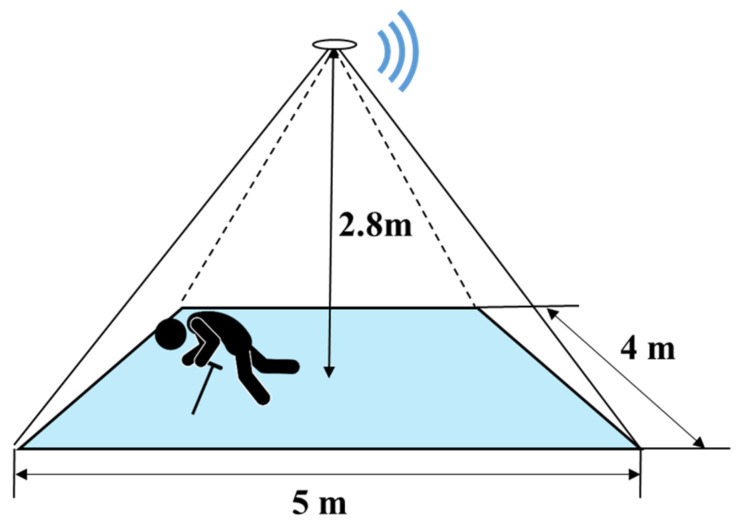
Diagram of experiment environment.

The sensor with a visual angle of 110° × 75° was attached to the ceiling of the room above the center of the detection area, as shown in Figure 11. The experiments were conducted in an area of which size is 4 m × 5 m. In addition, the experiment environment was complex as it contained chairs, desks, and computers, an accurate simulation of the actual application environment. As the figure shows, the height of the experiment room was 2.8 m, which is a common height of the intended application environments such as hospitals and nursing rooms.

During the experiments, the environment temperature was set around 25 °C, which was a comfortable condition for people and is common in real application conditions. All the participants were asked to make the target motions that were detected in the proposed motion detection system by following their habits.

### 4.2. Motion Dataset

In this motion detection research, there were eight target motions, as shown in Table 2, and each motion was recorded for 3 s to obtain motion frames. It was repeated 50 times to construct a dataset. Among them, the motion type ‘one motion’ means that this motion started at the beginning of the frames and finished by the end of the frames. The motion type ‘static motion’ means that this motion started at the beginning of the frames and kept the posture until the end with slightly movement. The motion type ‘continuous motion’ means that this motion continued from start to end of the frames with changing direction randomly.

A total of 16 participants’ data were collected, which means that 800 sets of data were collected for each motion. During the experiment, the participants were asked to perform corresponding actions in different directions in the detection area. For static movements, like sitting and standing, the participants were asked to hold for five seconds. For other dynamic actions, the participants were required to follow their habits. In all the motions, the experiments were performed many times in different positions of the detection area to ensure the diversity of data obtained throughout the experiments.

### 4.3. Experiment Ethics

The risk of experimenting with elderly people is prohibitively high. As the resolution of the infrared array sensor used in this system is relatively low, it can be supposed that the characteristics of the data acquired from young people are roughly similar to those of the elderly. Therefore, the participants in this study were all in their twenties.

It was required to obtain fall motion data from the experimenters, which might be dangerous. Therefore, the safety of the participants was considered. During the experiment, a sofa and a large sponge pad were prepared to protect the experimenters. 

All participants gave their informed consent for inclusion before they participated in the study. The study was conducted in accordance with the Declaration of Helsinki, and the protocol was approved by the Ethics Committee of H29-230.

## 5. Results

This section explains calculation setting and cross-validation results of two classifiers, selection of input frame number, computation time of training and testing for two classifiers, and application to multi-target detection.

### 5.1. Cross-Validations and Results

In this research, datasets from 16 participants were collected from the infrared area sensor. After gathering the data, a series of data processing methods such as noise reduction and background removal were performed. However, it is unclear how many frames of data can accurately represent a motion suitable for one detection. Hence, 15 frames were tentatively set to the sequential data length. The 15 frames extracted from the sequential frames were directly input into the 3D CNN to detect a motion. In the LSTM network, after calculating the six features, a 6 × 15 feature matrix was used for detection.

In this system, the parameter α of Equation (2) was set to 0.999 by considering the frame rate of the sensor, and the parameter $N_{H}$ of Equation (3) was set to 8 by considering the target size in the actual data. In the training processes of both classifiers, the learning rate was set to 0.001, and the number of maximum epochs was set to 100.

In order to improve the versatility and reliability of the system, multiple experiments were used to examine the effects of individual differences. The detection performance was evaluated using commonly used cross-validation [62]. The cross-validations were performed on data from 14 participants for training and data from two participants for the testing. This process was repeated eight times by changing the combination of two testing data set (8-fold cross-validations). For the experiments, the results and the correct answer rates of every motion under the LSTM network and 3D CNN are shown in Table 3 and Table 4, respectively.

Table 5 shows the average, maximum, and minimum correct answer rates of each motion for the 3D CNN cross-validation. 

### 5.2. Input Frame Namber Selection

**Figure 12:**
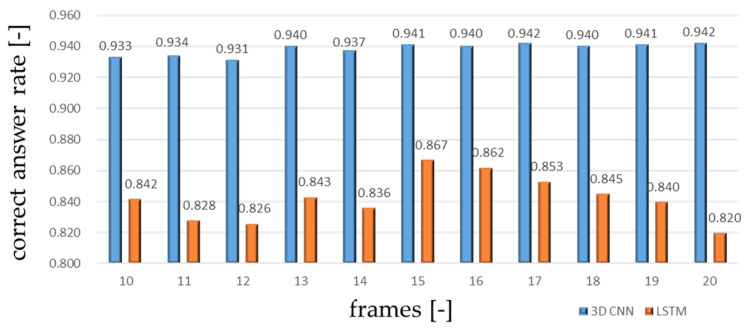
Correct answer rates of LSTM network and 3D CNN for each frame number.

A human motion is a continuous process involving a change of body status. From the observation of the motions in the experiment, all the motions other than ‘walking’ could take 0.7 to 1 s from the start to end of the motion. This means that, in the obtained data at 15 Hz sampling rate, the motion appeared within 15 frames. In order to confirm the proper input frame number between 10 and 20 frames were tested for comparison. The result of this experiment is shown in Figure 12. 

### 5.3. Computation Time

The computation time of each classifier at the 15 frames input data with and without a graphics processing unit (GPU) for the cross-validations in Section 5.1 is shown in Table 6. The calculations were executed on a laptop PC with an Intel i7-4710HQ CPU (max: 3.5 GHz) and an NVIDIA GF850M GPU. The LSTM network, with or without a GPU, was six to nine times faster than the 3D CNN.

### 5.4. Multi-Target Detection

**Figure 13:**
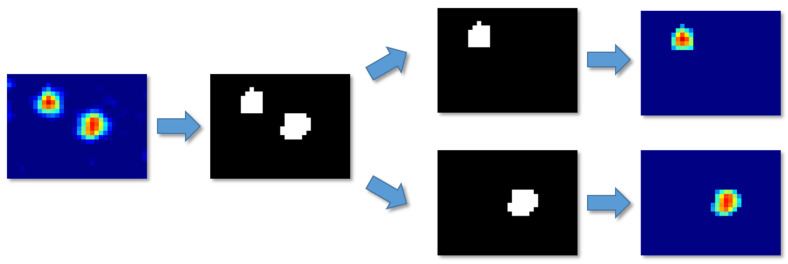
Multi-target detection.

In the actual application of the system, it is very likely that multiple targets will exist in the detection area at the same time. Therefore, by using the Otsu method, the number of targets can be counted from a binarized image. The areas of different targets in the image were divided into the different images with the background of the lowest temperature, and then motion detection was performed for each image as shown in Figure 13.

Data of two and three targets in the detection area were obtained to conform the capability of the system for multi-target situations. In total, 100 kinds of situations for two and three targets, in which one target fell while the others were walking, were processed and classified with the 3D CNN. The results are shown in Table 7.

## 6. Discussion

In this section, we discuss correct answer rates of motion detection in terms of for each motion and frame rates, performance metrics and comparison result with other studies, and limitation of the proposed system.

### 6.1. Ccorrect Answer Rates of Motion Detection

(1) Each motion detection

As seen in the results shown in Table 3, the average correct answer rate of the LSTM network for eight kinds of motions in this research was 82.5% for 16 participants. It can be concluded that the LSTM network performs well in the classification of the proposed eight motions, and especially for the motion of falling, the accuracy rate is over 92%. However, some of the correct answer rates could not reach 80%. The reason for these results is that the six extracted features cannot contain enough information for the classifier to distinguish the eight motions. As seen in the results in Table 4, the average correct answer rate for the 3D CNN of the eight motions in this research was 94.2% for the data of 16 participants. It can be concluded that the 3D CNN can perform better than the LSTM network in the classification of the eight motions, and automatically extract space and temporal information from the original image data. However, in some cases, the falling was misclassified as walking. The reason for these results is probably that among eight motions, falling and walking have more variety of movements than the others.

In Table 5, for each motion, the difference between the average and the minimum is obviously larger than the difference between the maximum and the average. The reason could be that a few participants’ motions were much different from the others’, resulting in a large part of the motion being divided into other motions. In order to avoid this problem, more data need to be collected to eliminate individual differences.

(2) Frame rates

According to the results of Figure 12, the test using 15 frames has a highest correct answer rate than the test using others in the LSTM network. For the 3D CNN, correct answer rates are close between 15 and 20 frames, those between 10 and 14 frames are slightly smaller. Therefore, considering the calculation time, it can be said that the setting of 15 frames is better than the others. Of course, it depends on the sampling rate, and thus it should be selected based on the target movement.

(3) Computation time

On the PC with a GPU, the LSTM network could provide full real-time motion estimation at speeds of 15 fps; meanwhile, the 3D CNN can realize 8 fps estimation. The calculation time required for the 3D CNN is obviously longer than that of the LSTM network. However, due to the low-resolution grayscale image used in this system, it does not affect the actual application process.

(4) Multi-target detection

As the number of targets increases, the accuracies of motion detection and participant number estimation decrease. The main reason for this result is that when the targets are close to one another, the system regards them as one lager target and makes a misjudgment. It is possible that if a higher-resolution sensor is used, the accuracy might increase; however, the privacy invasion problem is also considered. It becomes a trade-off between privacy and accuracy; therefore, a suitable sensor resolution and setting need to be considered.

### 6.2. Performance Metrics and Comparison

In order to compare our system to other studies, the effectiveness of the classifiers is confirmed by five metrics [63]. Each metric is expressed as follows:

(7) $$accuracy\;=\;\frac{TP+TN}{\;TP+FP+TN+FN\;}.$$

(8) $$recall\;=\;\frac{TP}{\;TP+FN\;}.$$

(9) $$specificity\;=\;\frac{TN}{\;FP+TN\;}.$$

(10) $$precision\;=\;\frac{TP}{\;TP+FP\;}.$$

(11) $$F1-measure\;=\;\frac{2recall\cdot precision}{\;recall+precision\;}.$$

where, TP, FP, TN and FN are true positives, false positives, true negatives, and false negatives, respectively. In these metrics, the seven motions other than falling are categorized into non-falling (negatives). Table 8 shows performance and characteristics of our proposed system and other studies using infrared sensors.

The recent research of [26] used a CNN with an LSTM network for fall detection using UWB and compared their proposed methods to other research with a CNN in the cross-validation results. The results of their methods showed better evaluation values of 95.78%, 98.04%, and 95.33% for accuracy, recall, and specificity, respectively.

In the research of [30] using pyroelectric infrared sensors for fall detection, their proposed method with a second-layer random forest had 92.51% mean accuracy. In the research of [31], which used infrared sensors and detected falling, the proposed method with an SVM showed evaluation values of 92.45%, 98.00%, and 95.14% for precision, recall, and F1-measure, respectively. In the research of [37], an infrared sensor with 32 × 32 resolution was used and could detect only falling and walking using pixel number threshold method, In the research of [38], using deep convolutional neural network, they could achieve high accuracy; however, because of the small number of participants, the availability of the method is unclear.

From the comparison results, even though the performance metrics of these studies cannot be compared directly due to different experiment settings, it can be said that our proposed method with a 3D CNN is sufficiently accurate and highly useful.

The research of [31] used very unique and interesting idea to realize motion detection using multiple low-resolution infrared sensors. Each sensor was connected to sensor network and the detection area was easily expanded in the system. However, to detect motions in an area of 3 × 3.75 m, they needed 20 sensor nodes, which leads to an increase in system construction cost. In contrast, our proposed system uses one device to detect motions in an area of 4 × 5 m, and we can expand the detection area by using several devices independently. When we need to watch a wide area, some devices can be located on the grid on the ceiling and all devices are connected with Wi-Fi. They can also be placed separately at the entrance of a room or in a dangerous place. This means that our system has high flexibility in the construction of a fall detection system.

### 6.3. Limitation of the System

After subtraction of background in a thermo image, in almost all cases, temperature distribution of a human is within 5 °C as shown in Figure 5. The noise equivalent temperature difference of this sensor is 0.1 to 0.25 K, which depends on a sampling rate. Therefore, we can detect the target human as a gradient object by using this sensor. However, when the height of the sensor to be attached on the ceil is changed to a certain extent, the pixel size of the target could be changed. It may cause decrease in detection accuracy. Provided that we use a higher resolution infrared sensor, the accuracy can be improved more; however, as mentioned above, the risk of privacy invasion should be considered at the same time.

When a heat source other than a human exists in the detection area and its temperature fluctuates frequently, the system may misidentify it as a human. Provided that the position of the heat source is known in advance, a masking process can be applied on the thermo image in the preprocessing to ignore the effect of the heat source.

## 7. Conclusions

In this research, a human motion detection system based on an infrared array sensor was developed for recognizing elderly people’s fundamental motions and detecting emergencies while protecting the privacy of people to be cared for. Compared with existing human motion detection systems, the proposed system was faced with fewer limitations and had a wide application. The six features were extracted considering the characteristics of human motions for the LSTM training. The 3D CNN was applied to motion detection and the input was time series image data.

During the human motion detection experiments, 50 sets of data for each motion in eight motions, including falling, standing, standing-to-sitting, sitting, sitting-to-standing, bowing, crouching, and walking of 16 participants were obtained, which means that 800 sets of data were collected for each motion.

In this research, the correct answer rates of the LSTM network and the 3D CNN were 82.5% and 94.2%, respectively. The results show that the proposed system with the 3D CNN has the ability to detect eight motions among 16 participants with high accuracy.

## Figures and Tables

**Table 1:** Comparison with other studies using infrared sensors.

Study	Year	IR Sensor (Resolution)	Number of Sensors	Activities	Participants	Method	Limitations
Luo [30]	2017	pyroelectric	5	falling, walking, standing, lying, transitional	3	random Forest	L1
Tao [31]	2012	1 × 1	20	falling, walking, tidying, watching TV, reading taking drinks, using PC, lying, sweeping	1	martingale framework	
Mashiyama [32]	2015	8 × 8	1	stopping, walking, sitting, falling	6	support vector machine	L1
Jankowski [33]	2015	distance	2	falling	2	NN	L1,L2,L3
						nonlinear principal component analysis NN	
Chen [34]	2015	16 × 4	2	falling	5	k-nearest neighbor	L1,L2,L3
Hayashida [35]	2017	8 × 8	1	falling	7	distance threshold	L1,L3
Taramasco [36]	2018	1 × 8	2	falling, walking, jogging, sitting, crouching, jumping, rotating	4	bidirectional-LSTM	L1 L2
						LSTM	
						gated recurrent unit	
Liang [37]	2018	32 × 32	1	falling, walking	-	pixel number threshold	L1 L2
Gochoo [38]	2018	16 × 16	3	standing, hand raising, squat, akimbo, crawl, toe touch, lying, open wide arms	4	deep convolutional neural network	L1 L2
Adolf [39]	2018	8 × 8	1	standing, sitting, lying	4	Inception v3 neural network	L1
Ogawa [40]	2020	4 × 16	2	falling, walking. lying	10	voting	
Liu [41]	2020	8 × 8	1	falling	8	random Forest	L1,L3

Note: the symbol ‘-‘ means the corresponding term is not indicated in the paper clearly; L1 means only one target human detection; L2 means when obstacles exist it cannot detect motions because sensor(s) are not mounted on the ceiling; L3 means only fall can be detected.

**Table 2:** Target motions in the motion detection system.

Motion Name	Repetitions	Motion Type
falling	50	One motion
sitting to standing (sit2stand)	50	One motion
standing to sitting (stand2sit)	50	One motion
sitting	50	Static motion
standing	50	Static motion
bowing	50	One motion
crouching	50	Static motion
walking	50	Continuous motion

**Table 3:** Result of motion detection with the LSTM network.

Real	Detected								Correct Answer Rate
	Falling	Sit2stand	Stand2sit	Sitting	Standing	Bowing	Crouching	Walking	
falling	**740**	3	21	6	8	4	1	17	92.5%
sit2stand	26	**724**	9	0	9	7	14	11	90.5%
stand2sit	37	1	**694**	38	5	3	19	3	86.8%
sitting	4	0	1	**626**	26	97	35	11	78.3%
standing	7	0	0	18	**630**	80	57	8	78.8%
bowing	5	3	1	26	16	**649**	98	2	81.1%
crouching	0	4	4	32	22	138	**593**	7	74.1%
walking	2	41	7	15	10	45	50	**630**	78.8%

**Table 4:** Result of motion detection with the 3D CNN.

Real	Detected								Correct Answer Rate
	Falling	Sit2stand	Stand2sit	Sitting	Standing	Bowing	Crouching	Walking	
falling	**748**	0	0	0	0	0	0	52	93.5%
sit2stand	0	**720**	0	28	4	0	0	48	90.0%
stand2sit	5	0	**793**	2	0	0	0	0	99.1%
sitting	0	0	0	**745**	51	0	0	4	93.1%
standing	0	0	0	19	**769**	0	0	12	96.1%
bowing	0	8	0	8	7	**721**	42	14	90.1%
crouching	0	0	0	23	0	5	**765**	7	95.6%
walking	23	4	0	0	0	0	6	**767**	95.9%

**Table 5:** The average, maximum and minimum correct answer rate with the 3D CNN.

Motion	Average	Maximum	Minimum
falling	93.5%	99%	84%
sit2stand	90.0%	96%	80%
stand2sit	99.1%	100%	93%
sitting	93.1%	96%	80%
standing	96.1%	99%	87%
bowing	90.1%	93%	82%
crouching	95.6%	98%	90%
walking	95.8%	99%	84%
total	94.2%	97.5%	85.0%

**Table 6:** Result of calculation time for 15 frames input data with/without a graphics processing unit (GPU).

	With GPU	Without GPU
LSTM network training	1.5 h	3 h
3D CNN training	2 h	5 h
LSTM network testing	0.02 sec	0.07 sec
3D CNN testing	0.12 sec	0.65 sec

**Table 7:** Result of multi-target detection with the 3D CNN.

Number of Participant(s)	Correct Answer Rate of Motion	Correct Answer Rate of Participant Number Estimation
1	92%	100%
2	84%	91%
3	72%	85%

**Table 8:** Comparison with other studies using infrared sensors.

Study	IR Sensor (Resolution)	Number of Sensors	Activities	Participants	Method	Recall (%)	Precision (%)	Specificity (%)	F1-Measure (%)	Accuracy (%)
[30]	pyroelectric	5	falling, walking, standing, lying, transitional	3	random Forest	-	-	-	-	92.51
[31]	1 × 1	20	falling, walking, tidying, watching TV, reading taking drinks, using PC, lying, sweeping	1	martingale framework	98	92.45	-	95.14	-
[32]	8 × 8	1	stopping, walking, sitting, falling	6	support vector machine	-	-	-	-	94.7
[33]	distance	2	falling	2	NN	89	86	-	-	-
					nonlinear principal component analysis NN	92	93	-	-	-
[34]	16 × 4	2	falling	5	k-nearest neighbor	95.3	-	90.8	-	93
[35]	8 × 8	1	falling	7	distance threshold	-	-	-	-	83–97
[36]	1 × 8	2	falling, walking, jogging, sitting, crouching, jumping, rotating	4	bidirectional-LSTM	93	-	93	-	93
					LSTM	89	-	93	-	91
					gated recurrent unit	85	-	89	-	87.5
[37]	32 × 32	1	falling, walking	-	pixel number threshold	-	-	-	-	95
[38]	16 × 16	3	standing, hand raising, squat, akimbo, crawl, toe touch, lying, open wide arms	4	deep convolutional neural network	98.2	98.3	99.7	98.1	99.5
[39]	8 × 8	1	standing, sitting, lying	4	Inception v3 neural network	45.6	-	86.2	-	-
[40]	4 × 16	2	falling, walking. lying	10	voting	-	-	-	-	97.8
[41]	8 × 8	1	falling	8	random Forest	96	96	-	96	-
ours	32 × 24	1	falling, sitting to standing, standing to sitting, sitting, standing, crouching, bowing, walking	16	LSTM	92.5	90.1	98.5	91.3	97.8
					3D CNN	93.5	96.4	99.5	94.9	98.8

Note: the symbol ‘-‘ means the corresponding term is not indicated in the paper clearly.
